# Antiproliferative and metabolic effects of metformin in a preoperative window clinical trial for endometrial cancer

**DOI:** 10.1002/cam4.353

**Published:** 2014-11-21

**Authors:** Kevin M Schuler, Brooke S Rambally, Megan J DiFurio, Brante P Sampey, Paola A Gehrig, Liza Makowski, Victoria L Bae-Jump

**Affiliations:** 1 Division of Gynecologic Oncology, Good Samaritan HospitalCincinnati, Ohio; 2 Department of Pathology and Laboratory Medicine, University of North CarolinaChapel Hill, North Carolina; 3 Pinehurst Pathology CenterPinehurst, North Carolina; 4 MetabolonResearch Triangle Park, North Carolina; 5 Division of Gynecologic Oncology, University of North CarolinaChapel Hill, North Carolina; 6 Lineberger Comprehensive Cancer Center, University of North CarolinaChapel Hill, North Carolina; 7 Department of Nutrition, University of North CarolinaChapel Hill, North Carolina

**Keywords:** Endometrial cancer, metabolomics, metformin, mTOR pathway

## Abstract

We conducted a preoperative window study of metformin in endometrial cancer (EC) patients and evaluated its antiproliferative, molecular and metabolic effects. Twenty obese women with endometrioid EC were treated with metformin (850 mg) daily for up to 4 weeks prior to surgical staging. Expression of the proliferation marker Ki-67, estrogen receptor (ER), progesterone receptor (PR), adenosine monophosphate-activated protein kinase (AMPK), and downstream targets of the mammalian target of rapamycin (mTOR) pathway were measured by immunohistochemistry. Global, untargeted metabolomics analysis of serum pre- and postmetformin treatment, and matched tumor, was performed. Metformin reduced proliferation by 11.75% (*P* = 0.008) based on the comparison of pre- and posttreatment endometrial tumors. A total of 65% of patients responded to metformin as defined by a decrease in Ki-67 staining in their endometrial tumors post-treatment. Metformin decreased expression of phosphorylated (p)-AMPK (*P* = 0.00001), p-Akt (*P* = 0.0002), p-S6 (51.2%, *P* = 0.0002), p-4E-BP-1 (*P* = 0.001), and ER (*P* = 0.0002) but not PR expression. Metabolomic profiling of serum indicated that responders versus nonresponders to treatment were more sensitive to metformin's effects on induction of lipolysis, which correlated with increased fatty acid oxidation and glycogen metabolism in matched tumors. In conclusion, metformin reduced tumor proliferation in a pre-operative window study in obese EC patients, with dramatic effects on inhibition of the mTOR pathway. Metformin induced a shift in lipid and glycogen metabolism that was more pronounced in the serum and tumors of responders versus nonresponders to treatment.This study provides support for therapeutic clinical trials of metformin in obese patients with EC.

## Introduction

The American Cancer Society estimates that nearly 50,000 new cases of endometrial carcinoma will be diagnosed in 2014 1. Obesity, diabetes, and insulin resistance are well-known risk factors that drive the development of endometrial cancer 2,3. Unfortunately, obesity is not only a risk factor for endometrial cancer, but also may be associated with an increased risk of death from this disease 4. Specifically, an association with all-cause mortality in endometrial cancer as a function of body mass has been found, with obese patients having a relative risk of death 2.5 times higher than their nonobese counterparts and morbid obesity carrying a relative risk of over six times that of patients with a normal body mass index (BMI) 5. Obesity is an epidemic in the United States and worldwide, with over 65% of the US population being overweight and 33% obese 6. The severity of obesity-associated endometrial cancer suggests that tumors arising in the obese state may display altered tumor biology (or changes in the microenvironment) that drive carcinogenesis, providing a unique opportunity to inhibit obesity-activated pathways as a therapeutic strategy.

Metformin is an antidiabetic medication from the biguanide class that is widely used as the first line treatment of type 2 diabetes. Epidemiological evidence suggests that metformin use lowers cancer risk and reduces cancer deaths among diabetic patients 7–10, including those with endometrial cancer 11–13. Metformin is believed to have both indirect and direct effects on tumor growth 7,8, and it is unknown which of these effects are most important for metformin's antitumorigenic benefits. Its indirect effects are likely to be due to inhibition of hepatic gluconeogenesis, resulting in an improvement in insulin sensitivity and a reduction of blood glucose and circulating insulin levels, which may lead to decreased growth factor-stimulated tumor growth 7,8. On a direct level, metformin may affect tumor growth by activation of adenosine monophosphate-activated protein kinase (AMPK), its intracellular target for antidiabetic effects, which leads to the regulation of multiple downstream signaling pathways that control cellular proliferation, including inhibition of the mammalian target of rapamycin (mTOR) pathway 7,8. Alterations in the mTOR pathway, including inactivating PTEN mutations and PIK3CA amplifications or activating mutations, are common in endometrial cancers 14,15. Consistent with these observations, our pre-clinical studies reveal that metformin inhibits cell proliferation in endometrial cancer cell lines through inhibition of mTOR signaling, and behaves as a chemosensitizer when combined with cytotoxic agents 16,17. Preoperative window studies of metformin in patients planning to undergo surgical resection of breast cancer have shown promising results in metformin's ability to reduce proliferation indices (i.e., Ki-67) and increase apoptosis 18–20. It is likely that women with endometrial cancer may also benefit from metformin's antiproliferative effects, especially in obese patients who may have elevated circulating insulin and glucose levels as well as activation of the mTOR pathway in their endometrial tumors. Thus, we conducted a preoperative window clinical trial of metformin in obese women with endometrial cancer to evaluate short-term effects on cell proliferation and assess potential molecular and metabolic biomarkers of treatment response.

## Materials and Methods

### Study design

After obtaining approval from the University of North Carolina at Chapel Hill (UNC-CH) Institutional Review Board (IRB# 11-0575), a prospective, open label, preoperative window study was conducted to evaluate the effects of metformin on the endometrium in obese women with endometrial cancer. The inclusion criteria for enrollment in this study were that patients had to have tumors of endometrioid histology, were between the ages of 18–75 years, and were obese with a BMI ≥30. Surgical intervention was required within 7–28 days of enrollment. Patients were excluded if they were diabetics on metformin or insulin (currently or in the past 6 months), had an elevated creatinine (>1.0) or AST/ALT (>38 or >48, respectively), had a history of alcoholism or B12 deficiency, were pregnant, had hormonal intervention within 4 weeks of evaluation, or had any other contraindications to metformin therapy.

**Figure 1:**
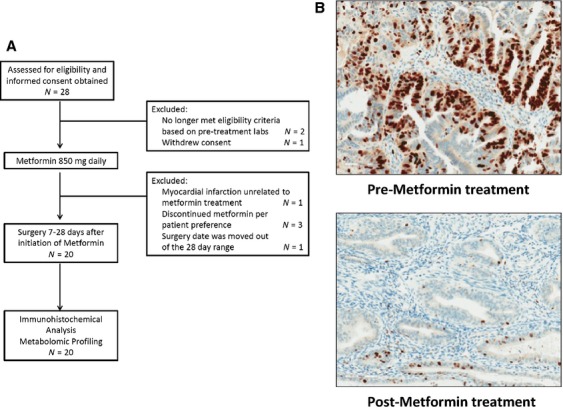
(A) Participant and trial flow diagram. (B) Metformin inhibited cellular proliferation in endometrial cancer patients. Obese endometrial cancer patients (*n* = 20) underwent short-term metformin treatment (mean of 14.65 days) in a preoperative window study. Percent Ki-67 staining, a marker of cellular proliferation, decreased significantly with metformin treatment (overall mean decrease of 11.75%, *P* = 0.008; mean decrease of 21.9% among responders to metformin.). Representative images are shown from pretreatment endometrium and posttreatment endometrium in a patient that responded to metformin treatment as demonstrated by a decrease in Ki-67 immunohistochemical staining.

The trial and participant flow diagram is described in Figure1A. Patient charts for all women presenting to the gynecologic oncology clinic at UNC-CH with a new diagnosis of endometrioid endometrial cancer were screened. Patients meeting these inclusion criteria were offered to participate in the study. If they wished to proceed, informed consent was obtained. Height and weight were recorded, and BMI was calculated. Laboratory evaluation, including creatinine, AST/ALT, and HgbA1c values, was obtained prior to metformin treatment. Once these values returned within treatment parameters, patients were started on metformin 850 mg orally daily. The research nurse for this study contacted the patients weekly after trial initiation to assess toxicity, using the definitions and criteria for grading provided in the NCI Common Toxicity Criteria for Adverse Events (CTCAE), version 4.0. Patients stopped metformin 24 h prior to surgery, as a means to decrease the rare but serious risk of lactic acidosis. Serum was collected and stored at −80°C pre- and post metformin treatment for metabolomic profiling. Patients were not fasting at the time the pretreatment serum specimens were collected. Patients were fasting at the time the posttreatment serum specimens were collected, given that these samples were collected at the time of hysterectomy and surgical staging. Since all patients fasted at the time of post-treatment collection of samples, differences observed between responders and nonresponders to metformin treated should not be affected by the fasting state. Fresh endometrial tumors were collected postmetformin treatment and snap frozen in liquid nitrogen for metabolomic profiling. Formalin-fixed, paraffin-embedded endometrial tumors from premetformin treatment endometrial biopsies and postmetformin treatment hysterectomy specimens were collected for immunohistochemical analysis. Pathologic characteristics of the endometrial tumors were obtained from pathology reports, including stage, grade, histology, and nodal status.

### Immunohistochemical analysis

Triplicate cores were made of endometrial tumors pre- (endometrial biopsy) and postmetformin treatment (hysterectomy), and tissue microarrays were created. Immunohistochemical analysis was performed on 4-*μ*mol/L sections of formalin-fixed, paraffin-embedded tissues using standard methodologies. The primary antibodies included the following: (1) anti-Ki-67 monoclonal antibody, M7240, Dako (Carpinteria, CA), (2) antiestrogen receptor (ER) monoclonal antibody, 249R-16, Cell Marque (Rocklin, CA), (3) antiprogesterone receptor (PR) monoclonal antibody, 323R-16, Cell Marque (4) antiphosphorylated AMPK*α* monoclonal antibody, 2535, Cell Signaling Technology (Danvers, MA), (5) antiphosphorylated Akt (ser 473) monoclonal antibody, 4060, Cell Signaling Technology, (6) antiphosphorylated S6 ribosomal protein monoclonal antibody, 4858, Cell Signaling Technology, and (7) antiphosphorylated 4E-BP-1 monoclonal antibody, 2855, Cell Signaling Technology. Negative controls (lacking primary antibody) were performed for each antibody. Individual slides were scanned using the Aperio™ ScanScope (Aperio Technologies, Vista, CA), and digital images were analyzed using Aperio™ ImageScope software. This work was performed with the assistance of the UNC-CH Translational Pathology Laboratory (TPL) Core.

### Metabolomic profiling

Metabolomic profiling was performed on serum obtained from patients pre- and postmetformin treatment as well as on endometrial tumors obtained post-treatment at surgery. Samples were analyzed by Metabolon (Research Triangle Park, NC) according to their standard protocols 21–24. Briefly, unbiased global metabolomic profiling was achieved using methanol extracts of serum or tumor tissues normalized to serum volume or tissue weight. Analysis of extracts consisted of either ultrahigh performance liquid chromatography (Waters Corporation, Milford, MA) coupled with tandem mass spectrometry (UHPLC/MS/MS; Thermo-Finnigan, San Jose CA) in positive and negative ionization modes, or via gas chromatography/MS analysis (Thermo-Finnigan). Metabolites in serum or tumor tissues were positively identified by matching chromatographic retention time, mass, and MS/MS fragmentation patterns to a reference library of over 2500 purified, authenticated biochemicals. Data are presented as relative measures of “scaled intensity” and median scaling to 1. Missing values were imputed with the minimum.

### Statistical analysis

The signed-rank test was used to evaluate the difference between pre- and posttreatment Ki-67, ER, PR, phosphorylated AMPK, phosphorylated Akt, phosphorylated S6, and phosphorylated 4E-BP-1immunohistochemical staining. Responders to metformin treatment were defined as those patients with an absolute reduction in %Ki-67 staining. Nonresponders were defined as those who had an increase in %Ki-67 staining. Demographics were compared between responders and nonresponders to metformin treatment, using the Student's *t*-test. Significance was defined at *P* < 0.05.

For the metabolomic profiling, two types of statistical analyses were performed: (1) significance tests and (2) classification analysis. For pairwise comparisons, Welch's *t*-tests and/or Wilcoxon's rank sum tests were performed. Where appropriate, repeated measures analysis of variance (ANOVA) were used. For classification analysis, random forest analyses were performed. Random forest is a supervised classification technique based on an ensemble of decision trees 25. For a given decision tree, a random subset of the data with identifying true class information is selected to build the tree (“bootstrap sample” or “training set”), and then the remaining data, the “out-of-bag” (OOB) variables, are passed down the tree to obtain a class prediction for each sample. This process is repeated thousands of times to produce the forest. The final classification of each sample is determined by computing the class prediction frequency (“votes”) for the OOB variables over the whole forest. Statistical analyses were performed with the program “R” (http://cran.r-project.org/).

## Results

### Study population

Pre- and postmetformin endometrial tumor specimens were obtained from 20 obese women with endometrial cancer (Fig.1A). The mean age was 58.8 years, and the mean pre-metformin treatment BMI was 39.6 kg/m^2^ (range 30.8–52.2 kg/m^2^). Patients received metformin for a mean duration of 14.6 days (range of 7–28 days) prior to surgical resection of the uterus. All patients had stage 1 or 2 disease, and 85% of the endometrial cancer tumors were either grade 1 or 2. Three patients experienced grade 1gastrointestinal toxicities while taking metformin, including abdominal pain, loose stools, and flatulence. These were all self-limited and did not require discontinuation of study drug. There was no grade 2 or higher toxicities among our study population, as defined by the NCI Common Toxicity Criteria for Adverse Events (CTCAE), version 4.0. Glucose levels decreased in responders and nonresponders to metformin but were only statistically significant in responders (*P* = 0.007).

### Metformin treatment prior to surgery reduced proliferation of endometrial tumors

Metformin significantly reduced Ki-67 staining by 11.75% in endometrial tumors, when comparing posttreatment hysterectomy specimens to pretreatment endometrial biopsies (*P* = 0.008) (Fig.1B). Responders to metformin treatment were defined as those patients with an absolute decrease in %Ki-67 staining (decrease range of 7–50%). Nonresponders were defined as those who had an increase in %Ki-67 staining (increase range of 2–12%). Overall, 65% of patients (13/20) responded to metformin treatment, with a mean decrease in Ki-67 staining of 21.9% among responders to metformin. No significant differences were detected in clinical factors (including age, premetformin treatment BMI, premetformin treatment HgbA1c, grade, stage, toxicity or number of days on treatment) between responders and nonresponders to metformin treatment (Table1). Pretreatment Ki-67 indices were statistically higher in women who responded to metformin treatment (47.3% vs. 24.9%, *P* = 0.004).

**Table 1:** Demographic information among responders and nonresponders to metformin treatment

Demographic	Responders (*N* = 13)	Non-responders (*N* = 7)	*P*-value
Age (years)	60 (9.6)	60 (9.6)	NS
BMI	38.4 (5.4)	41.8 (7.2)	NS
HgbA1c	5.6 (0.4)	5.6 (0.5)	NS
Duration of metformin treatment (days)	13.1 (5.3)	17.4 (7.7)	NS
Grade 1 AEs	1	2	NS
Grade 2-4 AEs	0	0	NS
Stage			
1A	8	7	NS
1B	4	0	NS
2	1	0	NS
Grade			
1	8	2	NS
2	4	3	NS
3	1	2	NS
Underwent nodal dissection	11	7	NS

AEs, adverse events.

### Metformin blunted mTOR signaling in tumors

**Figure 2:**
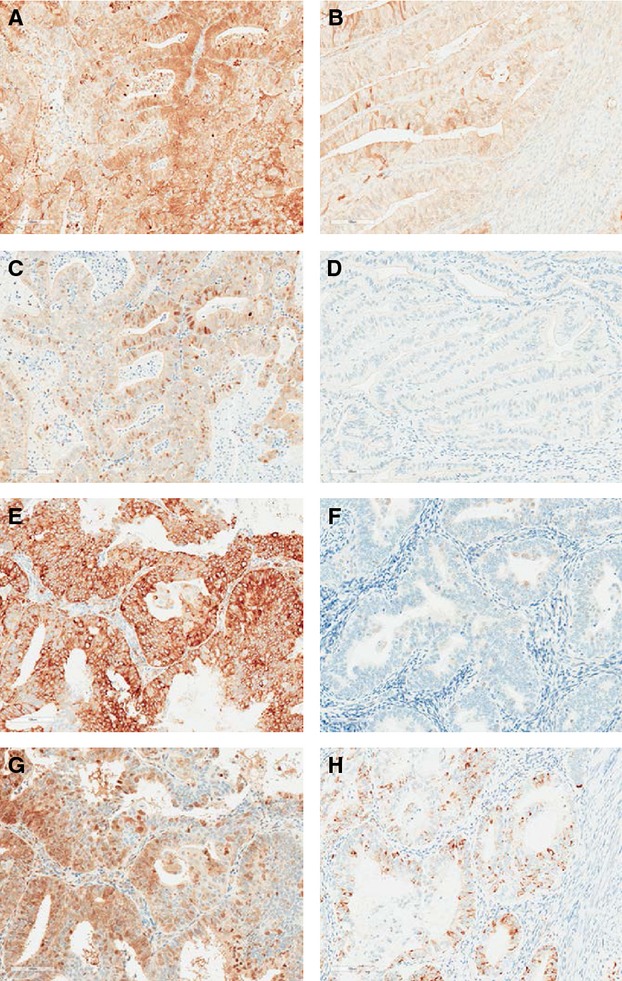
Metformin decreased expression of phosphorylated-Akt (*P* = 0.0002), phosphorylated-AMPK (*P* = 0.00001), phosphorylated-S6 (*P* = 0.0002),s and phosphorylated-4E-BP-1 staining (*P* = 0.001). (A) Phosphorylated-Akt staining premetformin treatment. (B) Phosphorylated-Akt staining postmetformin treatment. (C) Phosphorylated-AMPK staining premetformin treatment. (D) Phosphorylated-AMPK staining postmetformin treatment. (E) Phosphorylated-S6 staining premetformin treatment. (F) Phosphorylated-S6 staining postmetformin treatment. (G) Phosphorylated-4E-BP-1 staining premetformin treatment. (H) Phosphorylated-4E-BP-1staining postmetformin treatment.

Metformin significantly decreased phosphorylation of downstream targets of the mTOR pathway and impacted hormonal receptor expression in posttreatment hysterectomy specimens versus pretreatment endometrial biopsies. Metformin decreased expression of phosphorylated (p)-AMPK (60.3%, *P* = 0.00001), p-Akt (44.2%, *P* = 0.0002), p-S6 (51.2%, *P* = 0.0002), and p-4E-BP-1 (74.7%, *P* = 0.001) (Fig.2). ER expression was also decreased after treatment with metformin (65.7%, *P* = 0.0002); however, there was no effect on PR expression (*P* = 0.28). Pretreatment expression of ER, PR, p-AMPK, p-Akt, p-S6 or p-4E-BP-1 did not predict response to metformin therapy.

### Metabolic markers of respone to metformin treatment

We sought to determine the biochemical impact of metformin on patients with endometrial cancer by analyzing serum metabolomic profile changes from baseline to postmetformin therapy, specifically comparing responders versus nonresponders to metformin treatment. Serum was collected pre- and postmetformin treatment on 12/13 responders to metformin treatment and 6/7 nonresponders to metformin. Tumors were isolated for pathology and patient care, and the majority was available for metabolomics analysis. Postmetformin tumors were obtained from 9/13 responders and 3/7 nonresponders.

**Figure 3:**
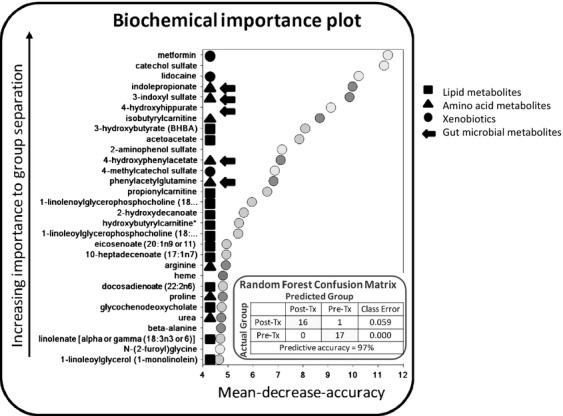
Random forest analysis of post-metformin treatment (Post-Tx) vs. premetformin treatment (Pre-Tx) serum samples from endometrial cancer patients. Random Forest classification using named metabolites in serum of Post-Tx compared to Pre-Tx sample gave an overall predictive accuracy of 97%, with a predictive accuracy of 91% for responders to metformin treatment versus only 67% for nonresponders to treatment. These changes were mainly related to alterations in lipid metabolism, amino acid, xenobiotic presence, and gut microbial-associated metabolites.

Metformin treatment significantly altered the serum concentrations of 173 metabolites (37 up and 136 down) (Table S1). Comparison of global biochemical profiles from serum and tumors revealed several key metabolic differences between responders and nonresponders to metformin treatment. In serum, 114 significant metabolites in responders and 67 metabolites in nonresponders were altered when compared to their respective premetformin treatment measures (Table S1). Although serum metformin appeared to be more elevated in the responder group than the nonresponder group posttreatment, a single outlier within the responder group drove this seeming difference that did not reach statistical significance (*P* = 0.7849). The observed difference in serum metformin between the two posttreatment groups is also likely complicated by levels of metformin approaching the limit of detection of the metabolomic platform in the fasted posttreatment groups, as metformin has been reported to be undetectable in human plasma by 24 h post-dose 26. Lidocaine showed the lowest *P*-value when comparing posttreatment groups versus pretreatment groups (Table S1), which is consistent with no lidocaine being detected in any pretreatment group sample and the perisurgical timing of the posttreatment sampling. Supervised classification showed a limited effect of metformin on nonresponder patients versus their respective baseline profiles, with a predictive accuracy of 67% by Random Forest analysis. In contrast, supervised classification demonstrated a predictive accuracy of 91% when comparing responders to their respective baseline profiles. The top thirty biochemicals that drove the ability to accurately classify samples from responders versus nonresponders are shown in Figure3. Metabolic alterations driven by metformin in the responders primarily related to elevated lipid metabolism, more efficient amino acid metabolism, increased xenobiotic presence, and altered gut microbiome-associated metabolites.

**Figure 4:**
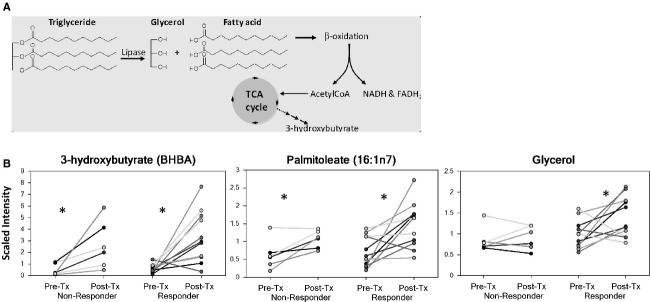
Lipid metabolism was altered in both responders and nonresponders to metformin treatment, but more pronounced effects were seen in the serum/ of responders. (A) Cartoon of triacylglyceride hydrolysis, *β*-oxidation, and tricarboxylic acid cycle (TCA cycle). (B) 3-hydroxybutyrate (BHBA), a marker of mitochondrial fatty acid *β*-oxidation, was elevated in both responders (Res) and nonresponders (Non-Res), but palmitoleate and glycerol were much more increased in responders to metformin treatment (*P < 0.05).

When comparing posttreatment to pretreatment serum, the greatest changes were seen in regard to lipid metabolism (Fig.4), with more significant effects observed in responders versus nonresponders to metformin treatment. Lipid metabolites increased by metformin treatment included ketone bodies, such as acetoacetate and 3-hydroxybutyrate, long chain fatty acids such as palmitoleate, 10-heptadecenoate, oleate, stearate, nonadecanoate, and eicosenoate as well as elevated glycerol levels (*P* < 0.05, Fig.4 and Table S2).

**Figure 5:**
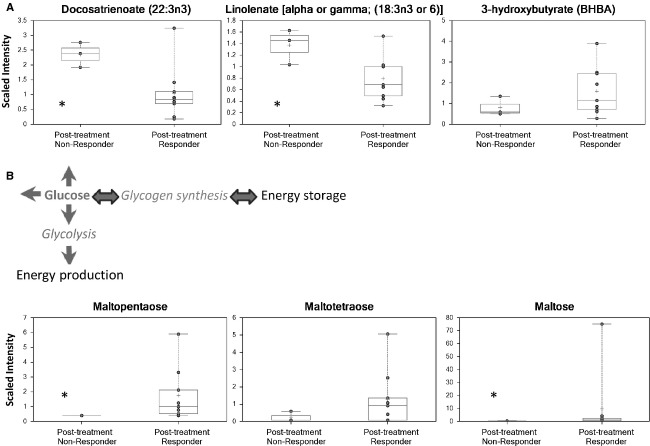
Metabolic changes in the endometrial tumors of responders as compared to nonresponders to metformin treatment were coincident with metabolic changes in the serum. (A) Several lipid metabolites, including docosatrienoate and linolenate, were decreased in the endometrial tumors of responders versus nonresponders to metformin treatment (*P* < 0.05). BHBA, a marker of mitochondrial fatty acid *β*-oxidation, was elevated in responder tissues but did not reach statistical significance (*P* = NS). (B) Increased glycogen synthesis was demonstrated in the tumors of responders as compared to nonresponders to metformin treatment. Glucose can be used for energy production through glycolysis or for storage through glycogen synthesis. Glycogen metabolites, maltopentaose (*P* < 0.05), maltotetraose (*P* = NS), and maltose (*P* < 0.05), accumulated in responders to metformin. *P < 0.05.

Metabolomic analysis of endometrial tumors after metformin treatment demonstrated significant differences in lipid metabolism in responders versus nonresponders (Fig.5). Fourteen metabolites related to lipid metabolism were found to be affected by metformin treatment, including polyunsaturated fatty acids and the ketone body, 3-hydroxybutyrate. Metformin was found to have differential effects in the endometrial tumors of responders versus nonresponders to treatment (Fig.5A, Table S2). In particular, docosatrienoate, linolenate, and dihomo-linolenate levels were lower in the endometrial tumors of responders versus nonresponders (*P* < 0.05). However, classification predictive accuracy was only 33% in separating endometrial tumors of responders versus nonresponders to metformin.

Glycogen synthesis was also significantly altered by metformin treatment in endometrial tumors. Glucose was elevated in the endometrial tumors isolated from responders as compared to nonresponders to metformin, although this was not statistically significant (Table S3). Elevated tumor glucose in the responder samples was concomitant with significantly elevated levels of several glycogen metabolites, including maltopentaose and maltose (*P* < 0.05) (Fig.5B), while maltotetraose was elevated but not statistically significant (Fig.5B).

## Discussion

In a preoperative window study in obese, nondiabetic endometrial cancer patients, we demonstrate that metformin significantly decreased proliferation in the malignant endometrium, with parallel effects on inhibition of the mTOR pathway. The majority of patients (65%) responded to metformin treatment as evidenced by a reduction in Ki-67 staining. Higher expression of baseline Ki-67 staining was a predictor of response to metformin therapy, suggesting that rapidly proliferating tumors responded best to this agent. Metformin was well-tolerated among the endometrial cancer patients enrolled, with no patients discontinuing treatment due to toxicities. Glucose levels decreased in responders and nonresponders to metformin but were only statistically significant in responders.

Differential effects of metformin were found in the serum of patients whose endometrial tumors responded favorably to drug exposure when compared to those who did not respond, as demonstrated by metabolomic profiling. The major overall biochemical response of patients to metformin treatment was related to lipid metabolism, with more significant effects seen in the serum of responders versus nonresponders to treatment. Lipid metabolites increased by metformin treatment included ketone bodies, long chain fatty acids, and glycerol. The most profound change in metabolite concentration in response to metformin was 3-hydroxybutyrate, a marker of mitochondrial fatty acid *β*-oxidation, which was significantly elevated in both responder and nonresponder groups. Elevations of free fatty acids and glycerol in the serum, most likely released primarily from adipose tissue, indicate that metformin-associated lipolysis was more pronounced in the responders versus nonresponders. The impact of metformin on lipolysis remains controversial with some studies reporting inhibition of lipolysis 27–31 and others reporting stimulation of lipolysis with metformin treatment in adipose tissue 32. Differences among these studies may be related to duration of treatment with metformin and method of detection of lipolysis and free fatty acids. Regardless, data presented herein suggest that endometrial cancer patients who responded to metformin were particularly sensitive to its metabolic effects on lipid metabolism, supporting the critical role of the indirect effects of metformin in endometrial cancer treatment. Of note, metformin was stopped 24 h prior surgery for safety reasons, i.e., minimizing the risk of lactic acidosis that could result from the stress of surgery. This may have some impact on the metabolomic profiling results; however, we would expect that the overall metabolic effects of metformin to persist beyond the 24 h of it being stopped prior to surgery.

Metabolic responses in the endometrial tumors of responders as compared to nonresponders to metformin treatment were coincident with metabolic changes in the serum. Despite the small sample size of endometrial tumors available for metabolomic analysis and the low predictive power in separating tumors from responders versus nonresponders (33%), 14 metabolites were identified that were differentially regulated by metformin response, including polyunsaturated fatty acids and the ketone body, 3-hydroxybutyrate. Lower free fatty acids and an increase in 3-hydroxybutyrate indicate greater fatty acid oxidation in tumors of responders to metformin treatment, which could also contribute to the apparent release of free fatty acids found in the serum. In addition, the most notable impact on lipid metabolites was with n3 and n6 fatty acids, which are known to serve as substrates for cyclooxygenase (COX)-mediated eicosanoid biosynthesis, which may indicate differential effects of metformin on tumor COX activity between responders and nonresponders to treatment.

**Figure 6:**
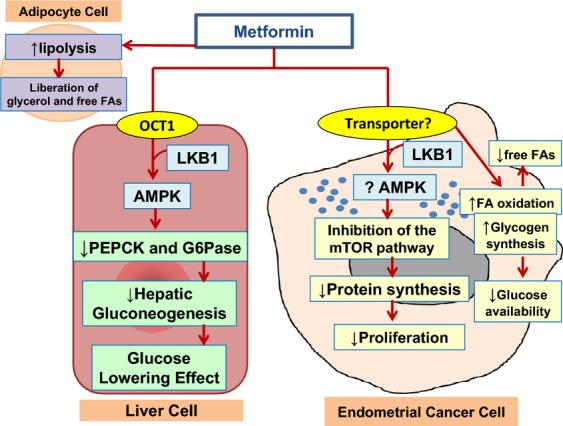
Indirect and direct effects seen by meformin in a preoperative window study in obese endometrial cancer patients. Metformin treatment resulted in a systemic decrease in serum glucose and enhanced lipolysis coupled with inhibition of the mTOR pathway and increased fatty acid oxidation and glycogen synthesis in the endometrial tumor cells themselves. These effects were more pronounced in responders versus nonresponders to metformin treatment.

Increased glycogen synthesis, as evidenced by a significant accumulation of several glycogen metabolites, was found in the tumors of responders versus nonresponders to metformin treatment. Energetically, this shift in glucose metabolism toward glycogen synthesis may result in diminished glucose availability to tumor cells. While in vitro studies have reported inhibition of glycogen synthesis in hepatocytes and myotubes 33,34, the acute concentrations of metformin that cells were exposed to in these studies were well above pharmacological relevance (200 *μ*mol/L–10 mmol/L vs. 10–20 *μ*mol/L reported pharmacologic concentrations) 35. In vivo studies, however, show that metformin treatment or chronic activation of AMPK via repeat dosing of rats with the adenosine analog 5-aminoimidazole-4-carboxamide ribonucleoside (AICAR) leads to glycogen accumulation in liver and muscle 36,37. Hence, the metabolomic profile of increased glycogen synthesis observed in endometrial tumors reported herein likely relates to dose- and duration-specific responses of endometrial tumors to metformin in vivo, when compared to in vitro exposure scenarios. Interestingly, lactate was not elevated in the tumor cells following metformin exposure, suggesting that metformin was not enhancing Warburg glycolytic metabolism, but instead was significantly shunting glucose within the tumor to storage as glycogen. Overall, systemic metabolic changes related to metformin treatment and response correlated to similar changes in the endometrial tumors themselves, signifying the interrelationship between the indirect and direct antitumor effects of metformin. In Figure6, we summarize the indirect and direct effects seen by meforminin in this preoperative window study, including a decrease in serum glucose and enhanced lipolysis coupled with inhibition of the mTOR pathway and increased fatty acid oxidation and glycogen synthesis in the endometrial tumor cells themselves. Of course, we do acknowledge that this study is limited by its small sample size, and our results need further validation in larger scale trials of metformin for endometrial cancer treatment. In addition, to further delineate the interaction between the metabolic and anticancer effects of metformin demonstrated in this clinical trial, parallel studies of metformin are underway in obese and nonobese endometrial cancer mouse models.

To date, three preoperative window trials of metformin in breast cancer patients have been conducted with mixed results. Of these studies, one was a single arm study, one was a randomized control trial of metformin versus no treatment, and one was a randomized controlled trial of metformin versus placebo. Two of these trials resulted in a statistically significant lowering of the Ki-67 index 18,19. BMI, weight and homeostasis model assessment (HOMA) scores also decreased significantly with short-term metformin treatment in the study by Niraula et al. 19. Transcriptome profiling of breast tumors pre- and postmetformin treatment found that metformin downregulated phosphodiesterase 3B, a critical regulator of cAMP levels that also regulates activation of AMPK 18. Metformin was also found to have significant effects on the tumor necrosis factor receptor-1, mTOR, AMPK, p53, BRCA1, and cell cycle pathways 18.

In the third and largest preoperative window study of metformin in breast cancer patients, Bonanni et al.*,* randomized 200 women to metformin or placebo in a 1:1 ratio 25. This study failed to reach its primary objective in reducing Ki-67 indices in postresection breast cancers 20. However, women with higher BMIs and HOMA indices had a significant response to metformin as evidenced by a decrease in Ki-67 staining 20. These findings suggest that the antitumorigenic effects of metformin may be more related to its ability to improve the metabolic milieu of patients as opposed to a direct action on tumor cells. Preclinical data in animal models also suggests that the antitumorigenic efficacy of metformin may be dependent on the metabolic composition of its host. Metformin has been found to be more effective in inhibiting tumor growth in obese and insulin resistant animals versus their lean counterparts in breast and lung cancer models 38,39. Thus, metformin may be more beneficial in those patients who are obese with insulin resistance, and further studies are warranted to determine whether the extent of obesity and the metabolic composition of the host may play a role in metformin's antitumorigenic effects.

There have been two other reported preoperative window studies of metformin in newly diagnosed endometrial cancer patients 40,41. As with the data presented here, endometrial cancer patients in both of these studies were treated with short-term metformin prior to hysterectomy and surgical staging 40,41. One of these studies demonstrated reduced Ki-67 staining in endometrial tumors postmetformin treatment 41 while the other found no effect 40. Serum insulin-like growth factor-1 (IGF-1) and leptin were found to decrease with metformin treatment in both of these studies 40. In the study by Soliman et al., metformin treatment resulted in decreased phosphorylation of Akt and MAPK in the malignant endometrium, with no effects on AMPK activation 40. Mitsuhashi et al. found that metformin resulted in decreased phosphorylation of S6 and the extracellular signal-regulated kinase 1/2 (ERK 1/2) and increased phosphorylation of AMPK.

Similar to the other preoperative window studies in breast and endometrial cancer 18,40, we found that metformin significantly decreased phosphorylation of downstream targets of the mTOR pathway, including p-Akt, p-S6, and p-4E-BP-1. Metformin was also found to decrease p-AMPK staining, which was counterintuitive to what we expected. It is known that metformin exerts its local antiproliferative effects through activation of AMPK; and thus, we would have expected an increase in AMPK phosphorylation instead of a decrease in endometrial cancer tumors with metformin treatment. Possible explanations for this observed finding could be related to an overall depletion in ATP as a result of metformin treatment or that metformin may not have direct effects on the endometrium itself. Controversy surrounds whether metformin's antitumorigenic benefits stems from its indirect effects via decreasing circulating insulin and glucose levels or its direct effects in tumor cells via AMPK activation and inhibition of the mTOR pathway. Our findings for evidence of mTOR pathway inhibition without AMPK activation in endometrial tissues could reflect a reduction in circulating growth factors such as insulin and glucose that indirectly leads to decreased activation of the mTOR pathway. Alternatively, metformin has also been found to inhibit the mTOR pathway via AMPK-independent mechanisms through its effects on the Ragulator complex (Rag GTPase) and REDD1 upregulation 42. Soliman et al. in their preoperative window study in endometrial cancer patients found that metformin had no effect on phophorylated-ACC, a substrate of AMPK 40. In addition, metformin did not increase AMPK signaling in obese rat endometrium, despite its robust effects in vitro 43.

Metformin was found to decrease ER expression in the malignant endometrium but had no effect on progesterone receptor expression. In endometrial cancer cell lines, as well as breast cancer animal models 44,45, metformin has been reported to increase progesterone receptor expression with little effects on ER expression. However, Markowska et al. reported in type 1 endometrial cancer specimens a decrease in ER expression but progesterone receptor expression, among the tumors of diabetic women on metformin versus those women using insulin 46. Metformin has been demonstrated to attenuate estrogen-stimulated proliferation in the obese rat endometrium and in normal rat endometrial cell lines 43. This most likely occurs via metformin's inhibitory effects on the mTOR pathway, regardless of whether this occurs by its hypothesized indirect or direct effects.

Strengths of our study include the use of a standard clinical starting dose of metformin that continued until 24 h prior to surgery. The utilization of metabolomic profiling to assess for metabolic biomarkers of response to metformin was a novel strategy embedded in this preoperative window clinical trial. Limitations of this study include the absence of a control or placebo arm, the small sample size, and the lack of posttreatment endometrial tumors on all patients enrolled. In addition, patients had a relatively short period of exposure to metformin, although the optimal duration of exposure to metformin for its potential antitumorigenic benefits is unknown.

Based on preclinical and epidemiological evidence, clinical trials are emerging for endometrial cancer and hyperplasia. Studies that are being conducted include a clinical trial of single agent metformin for the treatment of endometrial hyperplasia without atypia (NCT01685762), a chemoprevention study of metformin in obese women (NCT01697566), a phase 2 trial of metformin in combination with letrozole/RAD001 in advanced and recurrent endometrial cancer patients (NCT01797523), and metformin in combination with the levonorgestrel-releasing intrauterine device in nonsurgical patients with endometrial cancer/complex atypical hyperplasia (NCT02035787) (www.clinicaltrials.gov). Two of these trials are being conducted at our institution, and we plan to further assess the metabolites associated with response to metformin treatment in this preoperative window study through these other ongoing clinical trials. Lastly, the Gynecologic Oncology Group (GOG) is conducting a randomized, placebo-controlled phase 2/3 clinical trial of metformin in combination with paclitaxel/carboplatin versus paclitaxel and carboplatin alone in women with advanced and recurrent endometrial cancer (GOG 286B) (NCT02065687). This trial is uniquely stratified for obesity and should help answer the question of whether obesity and insulin resistance will predict responsiveness to metformin for cancer treatment, as some of the preclinical studies suggest 20,38,39. In addition to BMI, other metabolic characteristics will be followed throughout this trial, including hip-to-waist ratio and fasting insulin and glucose levels.

In conclusion, the preclinical, epidemiologic, and clinical data supporting the use of metformin in the prevention and treatment of cancers is building, including that of endometrial cancer. The association between obesity, insulin resistance, and increased risk and poor outcomes in endometrial cancer patients makes metformin an attractive agent for the prevention and treatment of this disease. Multiple clinical trials are in progress that will shed further light on the potential benefits of metformin in cancer patients, including that of endometrial cancer patients.

## Supporting Information

**Table S1.** Serum metabolites significantly altered in responders and nonresponders to metformin when comparing post- and pretreatment. Metabolomic analysis of serum indicated significant responses to metformin treatment in non-responders (Non-Res), responders (Res) and grouped posttreatment (Tx) over pretreatment (**P* < 0.05, #*P* = 0.1–0.05). Serum was successfully collected pre- and postmetformin treatment on 12/13 responders to metformin treatment and 6/7 nonresponders to metformin, and these samples were included in this metabolomic analysis.

**Table S2.** Lipid metabolites that were altered in the serum of endometrial cancer patients after treatment with metformin. Serum was successfully collected pre- and postmetformin treatment on 12/13 responders to metformin treatment and 6/7 nonresponders to metformin, and these samples were included in this metabolomic analysis.

**Table S3.** Lipid metabolites that were altered in the tumors of endometrial cancer patients after treatment with metformin. Postmetformin tumors were successfully obtained from 9/13 responders and 3/7 nonresponders.

**Table S4.** Glycogen metabolites that were altered in the tumors of endometrial cancer patients after treatment with metformin. Postmetformin tumors were successfully obtained from 9/13 responders and 3/7 nonresponders.
