# Supplementary material for: Antiproliferative and metabolic effects of metformin in a preoperative window clinical trial for endometrial cancer
Source: Cancer Med. 2014 Nov 21;4(2):161–73. doi: 10.1002/cam4.353 (PMC4329001; doi:10.1002/cam4.353)
Supplement: Supplementary file 1 [file cam40004-0161-sd1.docx]

| **Supplemental Table 1. Serum metabolites significantly altered in responders and non-responders to metformin when comparing post- and pre-treatment.** Metabolomic analysis of serum indicated significant responses to metformin treatment in non-responders (Non-Res), responders (Res) and grouped post-treatment (Tx) over pre-treatment (*p < 0.05, #p = 0.1 – 0.05). Serum was successfully collected pre- and post-metformin treatment on 12/13 responders to metformin treatment and 6/7 non-responders to metformin, and these samples were included in this metabolomic analysis. | | | | | |
| --- | --- | --- | --- | --- | --- |
|  |  | | |  |  |
|  | **Post-Tx Non-Res** | **Post-Tx Res** | **Post-Tx** |  | **Post-Tx** |
|  |  |  |  |  | **Pre-Tx** |
| **Biochemical Name** | **Pre-Tx Non-Res** | **Pre-Tx Res** | **Pre-Tx** | X |  |
| lidocaine | 6.51* | 16.41* | 12.92* |  | 2.84E-07 |
| catechol sulfate | 0.27* | 0.19* | 0.22* |  | 7.19E-07 |
| indolepropionate | 0.45* | 0.42* | 0.43* |  | 9.70E-07 |
| N-delta-acetylornithine* | 0.61* | 0.68* | 0.65* |  | 2.31E-06 |
| isobutyrylcarnitine | 0.52* | 0.47* | 0.49* |  | 9.52E-06 |
| urea | 0.65* | 0.74* | 0.71* |  | 1.10E-05 |
| arginine | 0.66* | 0.75* | 0.72* |  | 1.29E-05 |
| propionylcarnitine | 0.64* | 0.68* | 0.66* |  | 1.33E-05 |
| 3-hydroxypropanoate | 0.76# | 0.62* | 0.67* |  | 1.91E-05 |
| 4-hydroxyphenylacetate | 0.42* | 0.49* | 0.47* |  | 2.03E-05 |
| 3-indoxyl sulfate | 0.5* | 0.49* | 0.5* |  | 2.53E-05 |
| 2-aminooctanoate | 0.58* | 0.74* | 0.68* |  | 3.01E-05 |
| 3-hydroxybutyrate (BHBA) | 9.21* | 8.84* | 8.97* |  | 3.10E-05 |
| deoxycarnitine | 0.83* | 0.85* | 0.84* |  | 4.57E-05 |
| acetoacetate | 4.36* | 5.98* | 5.41* |  | 5.76E-05 |
| metformin | 4.98* | 9.72* | 8.05* |  | 5.79E-05 |
| 4-hydroxyhippurate | 0.67# | 0.46* | 0.54* |  | 8.12E-05 |
| phenylacetylglutamine | 0.66# | 0.46* | 0.53* |  | 9.87E-05 |
| indolelactate | 0.76# | 0.65* | 0.69* |  | 0.0001 |
| indoleacetate | 0.58* | 0.68* | 0.64* |  | 0.0001 |
| docosadienoate (22:2n6) | 1.50* | 1.73* | 1.65* |  | 0.0001 |
| 1-linolenoylglycerophosphocholine (18:3n3)* | 0.62# | 0.59* | 0.60* |  | 0.0001 |
| gamma-tocopherol | 0.64* | 0.74* | 0.70* |  | 0.0001 |
| pyridoxate | 0.55* | 0.57* | 0.56* |  | 0.0001 |
| proline | 0.77# | 0.78* | 0.78* |  | 0.0002 |
| arabitol | 0.79# | 0.76* | 0.77* |  | 0.0002 |
| glycocholate | 0.33* | 0.57* | 0.49* |  | 0.0002 |
| beta-alanine | 0.52* | 0.60* | 0.57* |  | 0.0002 |
| 3-hydroxyhippurate | 0.39* | 0.48* | 0.45* |  | 0.0002 |
| 3-methyl catechol sulfate 1 | 0.45# | 0.40* | 0.42* |  | 0.0002 |
| 4-vinylphenol sulfate | 1.09 | 0.25* | 0.55* |  | 0.0002 |
| ornithine | 0.74# | 0.68* | 0.70* |  | 0.0003 |
| 1-linoleoylglycerophosphocholine (18:2n6) | 0.67* | 0.70* | 0.69* |  | 0.0003 |
| glycochenodeoxycholate | 0.26* | 0.52* | 0.43* |  | 0.0003 |
| gamma-CEHC | 0.61# | 0.58* | 0.59* |  | 0.0003 |
| gluconate | 0.72# | 0.73* | 0.73* |  | 0.0003 |
| piperine | 0.48* | 0.59* | 0.55* |  | 0.0003 |
| 2-hydroxydecanoate | 0.61# | 0.49* | 0.53* |  | 0.0004 |
| quinate | 0.66 | 0.37* | 0.48* |  | 0.0004 |
| 2-aminophenol sulfate | 0.62# | 0.41* | 0.49* |  | 0.0004 |
| allantoin | 0.78 | 0.55* | 0.63* |  | 0.0005 |
| theobromine | 0.77 | 0.45* | 0.56* |  | 0.0005 |
| valine | 0.81# | 0.82* | 0.82* |  | 0.0006 |
| ascorbate (Vitamin C) | 0.73 | 0.48* | 0.57* |  | 0.0006 |
| 17-methylstearate | 1.36 | 1.92* | 1.72* |  | 0.0007 |
| N-methyl proline | 0.46* | 0.50* | 0.48* |  | 0.0008 |
| hydroxybutyrylcarnitine* | 3.13* | 4.28* | 3.87* |  | 0.0008 |
| 4-methylcatechol sulfate | 0.64# | 0.47* | 0.53* |  | 0.0008 |
| 1,3,7-trimethylurate | 0.63 | 0.55* | 0.58* |  | 0.0008 |
| tyrosine | 0.74# | 0.81* | 0.78* |  | 0.001 |
| palmitoleate (16:1n7) | 2.41* | 3.17* | 2.90* |  | 0.001 |
| palmitoyl ethanolamide | 1.25 | 1.57* | 1.46* |  | 0.001 |
| N-acetylthreonine | 0.88 | 0.73* | 0.78* |  | 0.0011 |
| alanine | 0.73# | 0.73* | 0.73* |  | 0.0011 |
| phosphate | 0.82* | 0.85* | 0.84* |  | 0.0011 |
| 10-heptadecenoate (17:1n7) | 2.38* | 2.85* | 2.68* |  | 0.0012 |
| isoleucylvaline | 0.62* | 0.74* | 0.70* |  | 0.0013 |
| threitol | 0.67* | 0.77* | 0.73* |  | 0.0013 |
| linolenate [alpha or gamma; (18:3n3 or 6)] | 1.93# | 2.79* | 2.49* |  | 0.0013 |
| oleate (18:1n9) | 1.79* | 1.86* | 1.84* |  | 0.0014 |
| palmitoyl sphingomyelin | 0.74* | 0.81* | 0.79* |  | 0.0014 |
| N-acetylcarnosine | 0.87 | 0.84* | 0.85* |  | 0.0015 |
| 1-linoleoylglycerol (1-monolinolein) | 1.94* | 1.98* | 1.97* |  | 0.0015 |
| dihydroorotate | 0.68* | 0.74* | 0.72* |  | 0.0016 |
| glycolate (hydroxyacetate) | 0.75* | 0.85* | 0.81* |  | 0.0018 |
| 9-methyluric acid | 0.66# | 0.67* | 0.67* |  | 0.0019 |
| heme | 12.76 | 7.92* | 9.63* |  | 0.0019 |
| 3-(4-hydroxyphenyl)lactate | 0.89 | 0.79* | 0.83* |  | 0.0026 |
| phenol sulfate | 0.86 | 0.63* | 0.71* |  | 0.0026 |
| erythronate* | 0.83 | 0.79* | 0.80* |  | 0.0026 |
| glycerol 3-phosphate (G3P) | 0.88 | 0.62* | 0.72* |  | 0.0026 |
| 7-alpha-hydroxy-3-oxo-4-cholestenoate (7-Hoca) | 0.58* | 0.83# | 0.75* |  | 0.0026 |
| inositol 1-phosphate (I1P) | 0.74* | 0.79* | 0.78* |  | 0.0029 |
| N-(2-furoyl)glycine | 0.99 | 0.45* | 0.64* |  | 0.0031 |
| pipecolate | 0.74 | 0.75* | 0.75* |  | 0.0033 |
| linoleate (18:2n6) | 1.63# | 1.77* | 1.72* |  | 0.0033 |
| eicosenoate (20:1n9 or 11) | 2.16* | 2.38* | 2.30* |  | 0.0034 |
| kynurenine | 0.86 | 0.79* | 0.81* |  | 0.0036 |
| N-acetylserine | 0.86# | 0.85* | 0.85* |  | 0.0037 |
| glucose | 0.87 | 0.87* | 0.87* |  | 0.004 |
| taurochenodeoxycholate | 0.34* | 0.92# | 0.72* |  | 0.0041 |
| tryptophan | 0.85 | 0.83* | 0.84* |  | 0.0043 |
| 10-nonadecenoate (19:1n9) | 2.33* | 2.49# | 2.43* |  | 0.0045 |
| alpha-tocopherol | 0.82# | 0.81* | 0.81* |  | 0.0046 |
| taurocholate | 0.50# | 0.96* | 0.79* |  | 0.0047 |
| cholesterol | 0.84* | 0.88# | 0.87* |  | 0.0048 |
| 2-methylbutyrylcarnitine (C5) | 0.81# | 0.78* | 0.79* |  | 0.005 |
| nonadecanoate (19:0) | 1.16 | 1.37* | 1.29* |  | 0.005 |
| N1-Methyl-2-pyridone-5-carboxamide | 0.7# | 0.77* | 0.74* |  | 0.0051 |
| mannitol | 7.67 | 14.08* | 11.82* |  | 0.0052 |
| cholate | 0.83 | 0.69* | 0.74* |  | 0.0053 |
| glycodeoxycholate | 0.45# | 0.71# | 0.62* |  | 0.0053 |
| 2-hydroxyisobutyrate | 0.91 | 0.79* | 0.83* |  | 0.0053 |
| androsterone sulfate | 0.73* | 0.87# | 0.82* |  | 0.0055 |
| N-acetylglycine | 1.79 | 1.56* | 1.64* |  | 0.0056 |
| 2-piperidinone | 0.76 | 0.72* | 0.73* |  | 0.0057 |
| erythritol | 0.84 | 0.82* | 0.83* |  | 0.0058 |
| epiandrosterone sulfate | 0.76# | 0.85# | 0.82* |  | 0.0062 |
| arabinose | 0.73* | 0.79# | 0.77* |  | 0.0063 |
| decanoylcarnitine | 0.64* | 0.79# | 0.74* |  | 0.0066 |
| 3-(3-hydroxyphenyl)propionate | 0.43* | 0.92 | 0.74* |  | 0.007 |
| glycoursodeoxycholate | 0.31* | 0.90 | 0.69* |  | 0.0072 |
| cis-4-decenoyl carnitine | 0.72# | 0.75* | 0.74* |  | 0.0074 |
| 2-hydroxyhippurate (salicylurate) | 0.48 | 0.61# | 0.56* |  | 0.0079 |
| stearate (18:0) | 1.24# | 1.43* | 1.36* |  | 0.0081 |
| 2-linoleoylglycerophosphocholine* | 0.65# | 0.83# | 0.77* |  | 0.0082 |
| lathosterol | 0.61* | 0.90 | 0.79* |  | 0.0083 |
| dihomo-linoleate (20:2n6) | 1.84* | 2.04 | 1.97* |  | 0.0087 |
| gamma-glutamylalanine | 0.84 | 0.78* | 0.80* |  | 0.0093 |
| 1-palmitoylplasmenylethanolamine* | 0.77 | 0.79* | 0.79* |  | 0.0093 |
| p-cresol sulfate | 0.73 | 0.89* | 0.83* |  | 0.0104 |
| octanoylcarnitine | 0.66* | 0.82# | 0.76* |  | 0.0107 |
| laurylcarnitine | 0.92 | 0.72* | 0.79* |  | 0.012 |
| 2-hydroxybutyrate (AHB) | 1.73* | 1.18 | 1.37* |  | 0.0122 |
| 1-myristoylglycerophosphocholine (14:0) | 0.76 | 0.79# | 0.78* |  | 0.0128 |
| N-formylmethionine | 0.94 | 0.83* | 0.87* |  | 0.013 |
| glycerol 2-phosphate | 0.86 | 0.77* | 0.8* |  | 0.0132 |
| asparagine | 0.68* | 0.90 | 0.82* |  | 0.0137 |
| hydrochlorothiazide | 0.72 | 0.69* | 0.70* |  | 0.014 |
| margarate (17:0) | 1.39* | 1.88# | 1.71* |  | 0.0143 |
| caffeine | 0.93 | 1.06* | 1.02* |  | 0.0149 |
| 2-oleoylglycerophosphoethanolamine* | 1.32 | 0.56* | 0.83* |  | 0.0164 |
| 2-hydroxyglutarate | 0.85# | 0.80# | 0.82* |  | 0.0172 |
| 1-oleoylglycerophosphoethanolamine | 0.79 | 0.86# | 0.84* |  | 0.0181 |
| threonine | 0.77 | 0.86# | 0.83* |  | 0.0184 |
| 1-methylhistidine | 0.54# | 1.05 | 0.87* |  | 0.0188 |
| gamma-glutamylvaline | 0.84 | 0.82# | 0.83* |  | 0.0189 |
| butyrylcarnitine | 0.78# | 0.91 | 0.86* |  | 0.0193 |
| taurodeoxycholate | 0.68 | 0.94# | 0.85* |  | 0.0194 |
| 3-methylxanthine | 0.49* | 0.97 | 0.80* |  | 0.0196 |
| 3-methyl-2-oxovalerate | 0.83 | 0.84# | 0.84* |  | 0.0197 |
| myristoleate (14:1n5) | 1.78 | 3.13# | 2.66* |  | 0.0197 |
| docosapentaenoate (n3 DPA; 22:5n3) | 1.64* | 1.67 | 1.66* |  | 0.0201 |
| 15-methylpalmitate (isobar with 2-methylpalmitate) | 1.11 | 1.68* | 1.48* |  | 0.0203 |
| kynurenate | 0.84 | 0.88# | 0.87* |  | 0.0207 |
| 2-myristoylglycerophosphocholine* | 0.82 | 0.78# | 0.80* |  | 0.0212 |
| palmitate (16:0) | 1.24 | 1.57# | 1.45* |  | 0.0218 |
| 2-hydroxyibuprofen | 0.72 | 0.76# | 0.74* |  | 0.0226 |
| isoleucine | 0.80# | 0.90 | 0.87* |  | 0.0231 |
| myo-inositol | 0.90 | 0.81# | 0.84* |  | 0.0231 |
| 1-eicosatrienoylglycerophosphoethanolamine* | 1.35 | 0.62* | 0.88* |  | 0.0235 |
| lysine | 0.89 | 0.89# | 0.89* |  | 0.0247 |
| glycerate | 0.77 | 0.86# | 0.83* |  | 0.0256 |
| N2,N2-dimethylguanosine | 0.58* | 0.96 | 0.83* |  | 0.0265 |
| homostachydrine* | 0.69* | 0.90 | 0.82* |  | 0.0269 |
| 1,5-anhydroglucitol (1,5-AG) | 0.94 | 0.89# | 0.91* |  | 0.0292 |
| guanosine | 1.15 | 0.88# | 0.98* |  | 0.0295 |
| 2-aminoheptanoate | 0.96 | 0.78* | 0.84* |  | 0.0297 |
| 1,11-undecanedicarboxylate | 0.89 | 0.82# | 0.85* |  | 0.03 |
| gamma-glutamyltyrosine | 0.91 | 0.85# | 0.87* |  | 0.0305 |
| 7-beta-hydroxycholesterol | 0.73# | 0.90 | 0.84* |  | 0.0312 |
| N-oleoyltaurine | 3.09# | 1.84 | 2.28* |  | 0.0316 |
| 1,3-dimethylurate | 0.69* | 0.94 | 0.85* |  | 0.0317 |
| paraxanthine | 0.71 | 1.11 | 0.97* |  | 0.0343 |
| carboxyibuprofen | 0.76 | 0.74# | 0.75* |  | 0.0344 |
| gamma-glutamylleucine | 0.87 | 0.90# | 0.89* |  | 0.036 |
| thymol sulfate | 1.07 | 0.69* | 0.82* |  | 0.0367 |
| 3-dehydrocarnitine* | 0.70* | 0.92 | 0.84* |  | 0.0376 |
| myristate (14:0) | 1.30 | 2.15# | 1.85* |  | 0.0389 |
| 1-palmitoylglycerol (1-monopalmitin) | 1.22 | 1.59# | 1.46* |  | 0.039 |
| pelargonate (9:0) | 0.89 | 0.80# | 0.83* |  | 0.0395 |
| N-acetyl-beta-alanine | 0.90 | 0.84# | 0.86* |  | 0.0433 |
| glutathione, oxidized (GSSG) | 3.49 | 4.05# | 3.85* |  | 0.0434 |
| erucate (22:1n9) | 1.12 | 1.64# | 1.46* |  | 0.0437 |
| erythrulose | 1.08 | 0.74* | 0.86* |  | 0.0438 |
| dehydroisoandrosterone sulfate (DHEA-S) | 0.83 | 0.92 | 0.89* |  | 0.0446 |
| glycerol | 1.10 | 1.58* | 1.41* |  | 0.0448 |
| ibuprofen | 0.76 | 0.82 | 0.80* |  | 0.0469 |
| cis-vaccenate (18:1n7) | 1.45 | 1.47 | 1.46* |  | 0.0474 |
| 1,6-anhydroglucose | 0.60* | 1.27 | 1.03* |  | 0.0476 |
| 1,2-propanediol | 0.83 | 0.87 | 0.85* |  | 0.0488 |
| beta-hydroxyisovalerate | 0.90 | 0.93 | 0.92* |  | 0.0492 |
| citrulline | 0.93 | 0.85# | 0.88* |  | 0.0492 |
