# Supplementary figures and images for: Antiproliferative and metabolic effects of metformin in a preoperative window clinical trial for endometrial cancer
Source: Cancer Med. 2014 Nov 21;4(2):161–73. doi: 10.1002/cam4.353 (PMC4329001; doi:10.1002/cam4.353)

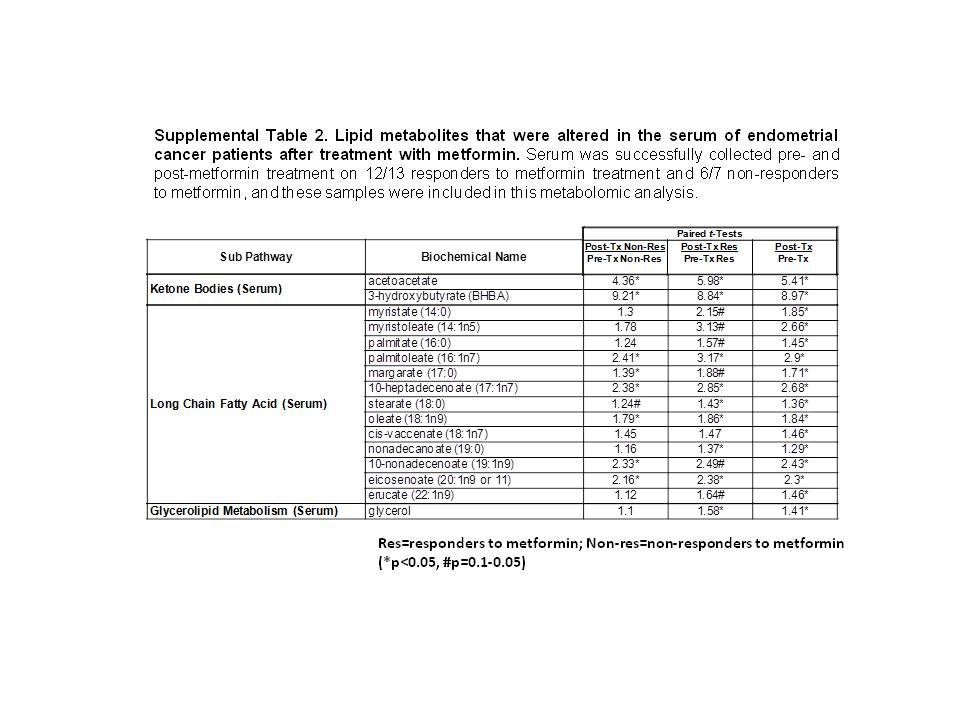

Supplement: Supplementary file 2 [file cam40004-0161-sd2.jpg]

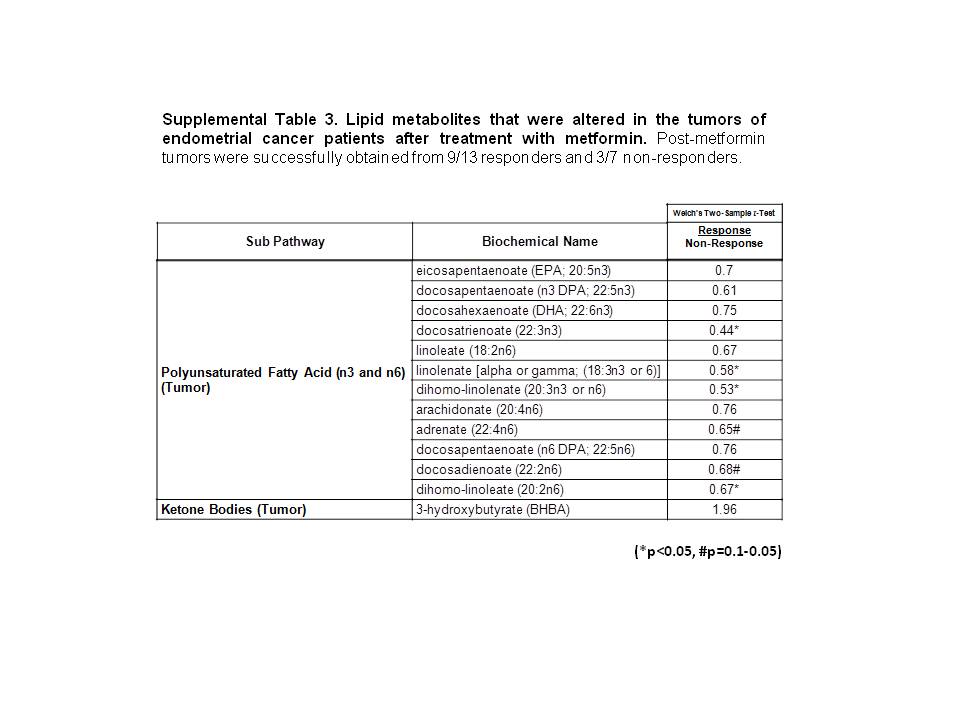

Supplement: Supplementary file 3 [file cam40004-0161-sd3.jpg]

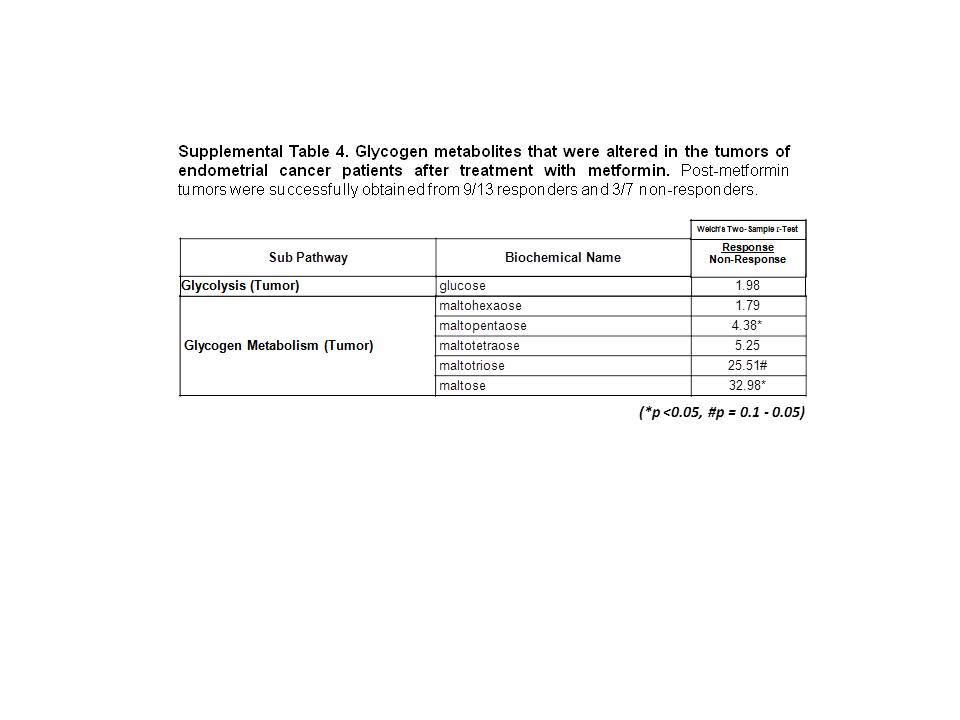

Supplement: Supplementary file 4 [file cam40004-0161-sd4.jpg]
